# Adapting a weight management tool for Latina women: a usability study of the Veteran Health Administration’s MOVE!23 tool

**DOI:** 10.1186/s12911-016-0368-2

**Published:** 2016-10-05

**Authors:** Hector R. Perez, Michael W. Nick, Katrina F. Mateo, Allison Squires, Scott E. Sherman, Adina Kalet, Melanie Jay

**Affiliations:** 1 Division of General Internal Medicine, Montefiore Medical Center, Bronx, NY 10467 USA; 2 Program for Medical Education and Technology, NYU School of Medicine, New York, NY 10016 USA; 3 Research, VA NY Harbor Healthcare System, 423 East 23rd Street, 15161N, New York, NY 10010 USA; 4 Medicine, NYU School of Medicine, New York, NY 10016 USA; 5 NYU College of Nursing, New York, NY 10003 USA; 6 Population Health, NYU School of Medicine, New York, NY 10016 USA

**Keywords:** Obesity, Usability testing, Qualitative research, Latino, Intervention research, Behavioral medicine

## Abstract

**Background:**

Obesity disproportionately affects Latina women, but few targeted, technology-assisted interventions that incorporate tailored health information exist for this population. The Veterans Health Administration (VHA) uses an online weight management tool (MOVE!23) which is publicly available, but was not designed for use in non-VHA populations.

**Methods:**

We conducted a qualitative study to determine how interactions between the tool and other contextual elements impacted task performance when the target Latina users interacted with MOVE!23. We sought to identify and classify specific facilitators and barriers that might inform design changes to the tool and its context of use, and in turn promote usability. Six English-speaking, adult Latinas were recruited from an inner city primary care clinic and a nursing program at a local university in the United States to engage in a “Think-Aloud” protocol while using MOVE!23. Sessions were recorded, transcribed, and coded to identify interactions between four factors that contribute to usability (Tool, Task, User, Context).

**Results:**

Five themes influencing usability were identified: Technical Ability and Technology Preferences; Language Confusion and Ambiguity; Supportive Tool Design and Facilitator Guidance; Relevant Examples; and Personal Experience. Features of the tool, task, and other contextual factors failed to fully support participants at times, impeding task completion. Participants interacted with the tool more readily when its language was familiar and content was personally relevant. When faced with ambiguity and uncertainty, they relied on the tool’s visual cues and examples, actively sought relevant personal experiences, and/or requested facilitator support.

**Conclusions:**

The ability of our participants to successfully use the tool was influenced by the interaction of individual characteristics with those of the tool and other contextual factors. We identified both tool-specific and context-related changes that could overcome barriers to the use of MOVE!23 among Latinas. Several general considerations for the design of eHealth tools are noted.

## Background

Obesity is associated with higher mortality [1] and an increased risk for multiple co-morbidities [2–5]. Developing targeted interventions to address obesity is important as some population groups have a high burden of obesity and may have unique health preferences [6–9]. Latina women are one such group, with about one-half with obesity, largely due to a combination of cultural, social, and genetic factors [10]. Research shows that Latinas have unique lifestyle patterns which influence their preferences about obesity management [11]. Nonetheless, despite the need for targeted interventions to address obesity among Latina patients, there are few developed specifically for them [12–14], and there are few robust studies testing eHealth interventions in this population [15]. In the face of this challenge, adapting existing obesity interventions to target Latinas is a viable option [16].

The MOVE! program is an evidence-based intensive weight management intervention created for Veterans by the Veterans Health Administration (VHA) available at all Veterans Affairs (VA) Medical Centers [17]. Before beginning the MOVE! program, patients complete the MOVE! online assessment tool (referred to as MOVE!23 at the time of the study as it contained 23 items) [18]. The MOVE! assessment tool collects information about the patient’s medical history, eating habits, level of physical activity, and potential barriers to weight management. Based on the patient’s responses, a customized summary report is generated that provides individualized advice supplemented by links to specific educational MOVE! materials [19]. After reviewing this report with the patient, MOVE! staff can then assist the patient with setting weight management-related goals and can encourage the use of other MOVE! resources such as group sessions.

Adaptation of the MOVE! assessment tool for use among Latina women may provide a cost-effective approach to improve weight management assessment and goal formation in this underserved population. The MOVE! assessment tool has been used to assess thousands of individuals enrolling into the MOVE! program, including female, urban-dwelling, minority Veterans [20]. The MOVE! program has also been associated with significantly greater 6-month weight loss outcomes in enrolled patients compared to unenrolled patients [21]. The MOVE! assessment tool is free and publically available online (now called MOVE!11) as are links to a library of over 100 educational weight management materials [19]. Although designed for use among Veterans, the VHA has suggested using it and the accompanying MOVE! materials among non-VA patients as well [18].

**Fig. 1:**
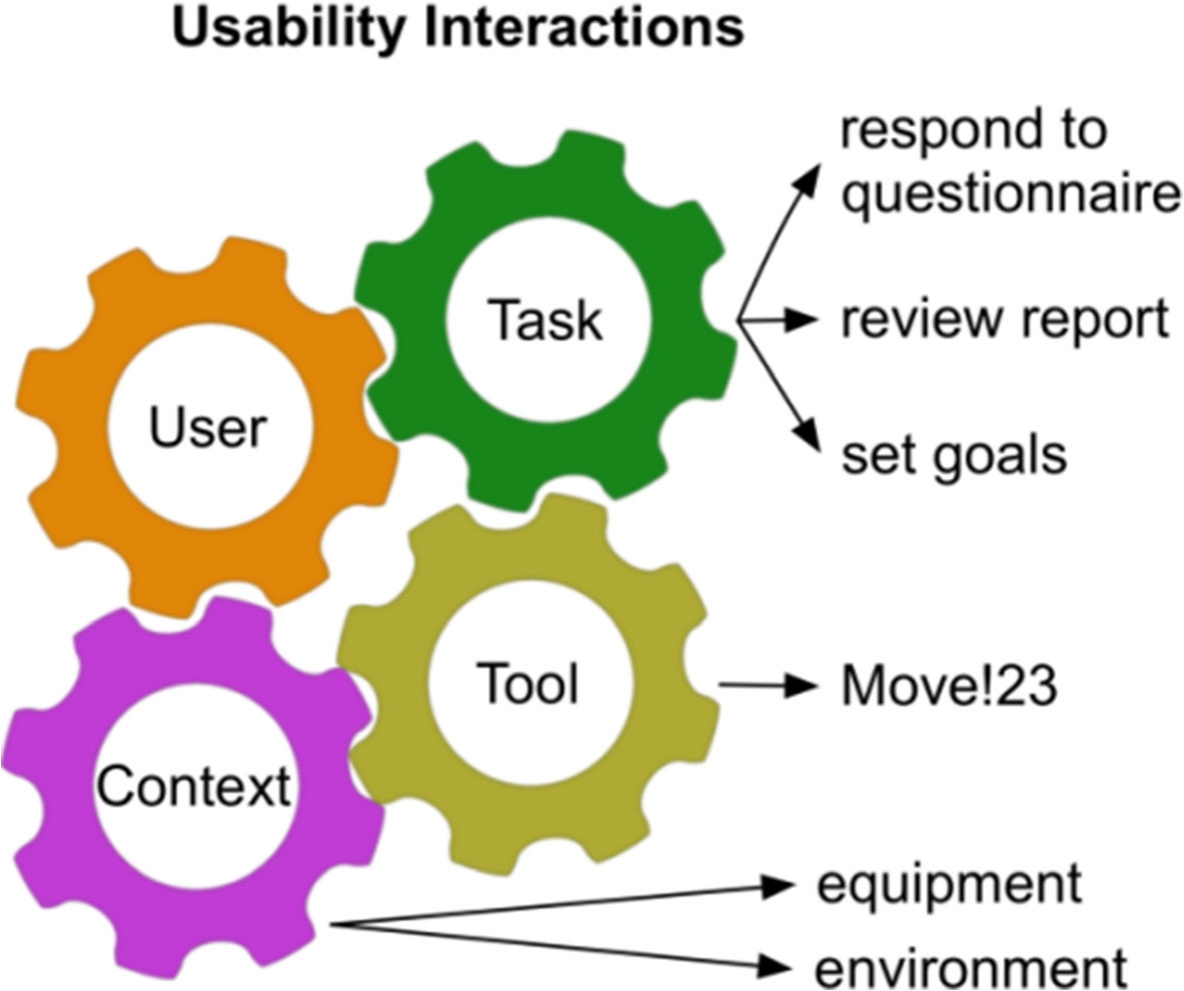
Factor interactions influencing usability. Colored gears represent four different usability factors (user, tool, task, context), and arrows specify the factor in the context of assessing the MOVE!23

Adapting the MOVE! tool to Latina women alters its context of use and can present unforeseen challenges [22]. The first step in the adaptation process is to determine whether patients in a new target population can successfully interact with the tool without confusion or frustration. Any impediments can reduce the tool’s effectiveness. Usability testing is a systematic method for determining the extent of interactions with a given tool. The International Organization for Standardization (ISO) has defined usability as the degree to which a tool promotes efficiency, effectiveness, and satisfaction as it is employed in the pursuit of a specific goal in a given context of use [23]. This ISO usability framework identifies several components whose individual characteristics and interactions impact usability: the user (“user”), the tool itself (“tool”), the task that is to be performed (“task”), and other contextual factors including equipment and the physical environment in which the tool is used (“context”). Figure 1 represents how we conceptualized usability interactions in this study. When the characteristics of these components are complementary, a task may be more readily accomplished. Conversely, when component characteristics are incompatible, tasks are more difficult to accomplish [24].

As a first step to adapt the MOVE! assessment tool, we conducted a qualitative usability study to better understand Latina users’ reactions to and interactions with the tool, the task itself, and other elements in the context of use. Our findings helped to identify and categorize changes that might improve interactions with the MOVE! tool. Lessons learned during the process may also inform the design of other eHealth interventions.

## Methods

### Recruitment

We sought to recruit between 4 and 8 participants for the usability evaluation of MOVE!23 to satisfy the studies’ objectives, operational limitations, and adhere to standard usability study practices [20, 21, 25, 26]. The number of participants was also purposely limited as this stage of the study was not intended to be an exhaustive evaluation of all usability issues associated with Latinas’ use of MOVE!23, to establish the efficacy of the MOVE! program, or to quantitatively evaluate MOVE!23 against similar assessment tools.

We used a combination of several convenience sampling approaches to recruit participants for this study. We recruited four participants from a pool of Latina patients that took part in a previous focus group study examining weight loss management and experiences [27]. Two additional participants were recruited from a nursing program at a local university. As MOVE!23 was available only in English, the ability to read, write, and converse in English was an inclusion criteria during recruitment. Given the project’s aims, we did not specifically recruit for certain sociodemographic characteristics during the recruitment process. All methods were approved by the Institutional Review Board at the New York University School of Medicine.

### Study procedure

An individual usability session in a private room was scheduled for each participant. Each participant provided written informed consent and completed a pre-study survey in English. The pre-study survey collected sociodemographic characteristics and measured health literacy [28]. MOVE!23 was accessed online using the Safari web browser on a 15-inch MacBook Pro. Participants had the option of using a standard mouse if they found the built-in trackpad to be cumbersome. Participants first completed the online questionnaire portion of MOVE!23, then reviewed the online tailored patient report created based on their responses with the session facilitator (MJ or MN). Printed copies of the patient report were made available, if requested, at the end of the session. Session facilitators did not have established relationships with the patients, were proficient in the use of the computer, and did not identify as Latino. No one else was present during each usability session.

We captured participants’ perceptions and judgments while interacting with MOVE!23 using a “Think-Aloud” protocol. This cognitive interviewing technique is a common usability evaluation method which allows researchers to gain insight into participants’ cognitive strategies and processing during problem-solving activities [24, 29–31]. Immediate verbalizations while interacting with the tool are believed to describe participants’ cognitive responses to a situation more accurately than retrospective interviews [32]. Participants were asked to verbalize their reactions and thoughts while responding to the questionnaire portion of the assessment tool, using its navigation features, and reviewing the tailored patient report. The session facilitator used verbal prompts to stimulate participant verbalizations. These prompts were often task focused (e.g., “What do you think of this handout?”), but became more feature specific to elicit clarification when verbalizations were unclear (e.g., “What do you think about the answer choices?”). The facilitator provided support when participants were unsure how to proceed with the task (e.g., “I don’t know how to use this thing”). For the purposes of this study, the task was defined as successful completion of the MOVE!23 questionnaire and review of the resulting tailored advice.

The facilitator conducted a semi-structured interview at the end of each session to confirm participants’ reactions during task performance. Interactions with MOVE!23 were recorded using the ScreenFlow screen capture software (version 4.0.3). Audio-recordings of each session were professionally transcribed. Transcripts were de-identified by research team members prior to analysis.

### Data analysis

Prior to analyzing the transcripts, we developed a three-tier coding scheme guided by the ISO usability framework to categorize participant’s utterances (see Table 1). This approach is similar to directed content analysis [33]. Six first-tier codes were assigned to utterances that reflected interactions between four elements of the usability scenario: tool-user, tool-task, tool-context, user-task, user-context, context-task. Three second-tier codes captured the impact that the interaction of existing factors had on task accomplishment (facilitates/impedes), or a participant’s suggestion to alter the interaction in some manner (wants). Facilitating interactions were those which promoted or enabled task accomplishment, while impeding interactions where those which hampered or prevented task accomplishment. The primary coder (MN) analyzed two transcripts, using semantic boundaries to define segments of text [34] and assigned fifty-seven third-tier codes to describe the main characteristic that appeared to underlie the verbalization (e.g. recollection of a past experience, language use in a question stem, function of tool component). A second coder (HP) applied this initial coding scheme to the same two transcripts to determine if the coding vocabulary was sufficient, and the coding scheme was refined accordingly. All six transcripts were then analyzed by both coders using this final codebook, which included a total of 216 unique codes. The two coders and a neutral third-party mediator (KM) met to review coded transcripts and negotiate any coding conflicts. The final coded segments were imported into the R statistical software package [35] for descriptive analyses, working from a summative content analysis approach where quantifying code frequency is part of the analytic process [33]. All coded statements were considered in synthesizing themes. Code frequencies were analyzed to help identify trends in the data and particular codes of interest. These supplemented discussions among the two coders and other research team members around themes and interpretations.

**Table 1:** Examples of coded transcript quotes based on three-tier coding scheme

Interaction/Code example	Example quote
Tier 1 Code: User/Tool Tier 2 Code: Facilitates Tier 3 Code: Patient Report, Relevance *(The subject makes a statement regarding the extent to which the patient report is personally relevant)*	RESPONDENT: I feel that, for the one with the foods, that I don’t ask enough. Like how it’s prepared…if I have it [patient report] with me at a restaurant, I would try to see how it’s prepared…It kind of made me think about that.
Tier 1 Code: User/Tool Tier 2 Code: Impedes Tier 3 Code: Question, Language *(The subject indicates that the language used by the survey question(s) is clear, appropriate, familiar to this user)*	RESPONDENT: Okay, I don’t understand this one “some form of dieting that is eating different from the way you usually eat for the sake of losing weight.” MODERATOR: Is that question difficult? Is that answer difficult to understand? RESPONDENT: That’s difficult to understand, yeah.
Tier 1 Code: User/Tool Tier 2 Code: Facilitates Tier 3 Code: Experience, Eating *(The subject reflects upon a personal experience related to eating habits when completing the questionnaire)*	RESPONDENT: When I’m focusing on the test or exam, I consume a lot of coffee, and then I could feel palpitations. So when I stop drinking a lot of coffee it stops so I have to say yes, I have too much stress right now.
Tier 1 Code: User/Context Tier 2 Code: Impedes Tier 3 Code: Technology, Experience *(The subject indicates that their experience/inexperience with a technology may impact their ability to “better” accomplish the task)*	RESPONDENT: I guess I’m used to PCs more and I always look for the scroll bar on the side. MODERATOR: You know, you can also go like this. Two fingers [motions on the touchpad]. RESPONDENT: Oh. Okay.

## Results

### Participant demographics

Table 2 includes demographics of the 6 study participants. All participants were female and the group had a mean age of 39.2 (SD 18.9, range 21–62). Four participants indicated that they were born in the continental United States, while two were born in Puerto Rico. Four were of Puerto Rican descent, one was of Ecuadorian descent, and one had mixed heritage from Nicaragua and Puerto Rico. Half of the participants completed college, and more than half indicated that English was spoken at home. Five out of the 6 participants indicated that they never required help reading health materials. Nonetheless, based on the Chew single-item health literacy screener [28], four participants indicated they lacked confidence in filling out health forms at least some of the time and, as such, were considered at risk for limited health literacy.

**Table 2:** Participant responses from pre-usability session survey (n = 6)

Participant ID	S-1	S-2	P-1	P-2	P-3	P-4
Gender	Female	Female	Female	Female	Female	Female
Age at time of study	22	21	57	24	49	62
Country of Origin	United States	United States	United States	United States	Puerto Rico	Puerto Rico
Length of time living in NYC	22	21	55	23	43	59
Primary language(s) spoken at home	English	Spanish	English & Spanish	English & Spanish	English	English & Spanish
Marital status	Single	Single	Married	Single	Separated/Divorced	Married
Level of school completed	College	College	Less than High School	Less than High School	College	Less than High School
How confident are you filling out medical and health forms by yourself?^a^	Quite Confident	Somewhat	Somewhat	Somewhat	Quite Confident	Not at all
Are you employed?	Yes	No	No	No	Yes	No

*Abbreviations*: *S* usability study student participant, *P* usability study patient participant

^a^Health Literacy Screener (possible responses: Not at all, Somewhat, Quite Confident, Extremely) [28]

### Transcript summary

**Fig. 2:**
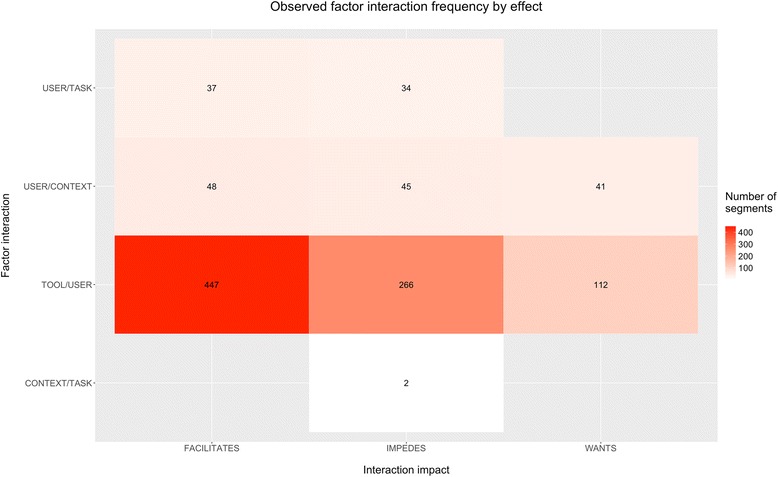
Frequency of observed factor interactions by interaction impact. Each coded segment is counted equally and is only listed in one group. The interaction impact represents whether the segment was coded positively (facilitates), negatively (impedes), or as a recommendation for improvement (wants). The number listed in each group is the total number of coded segments in that group. Blank boxes had zero coded segments

**Fig. 3:**
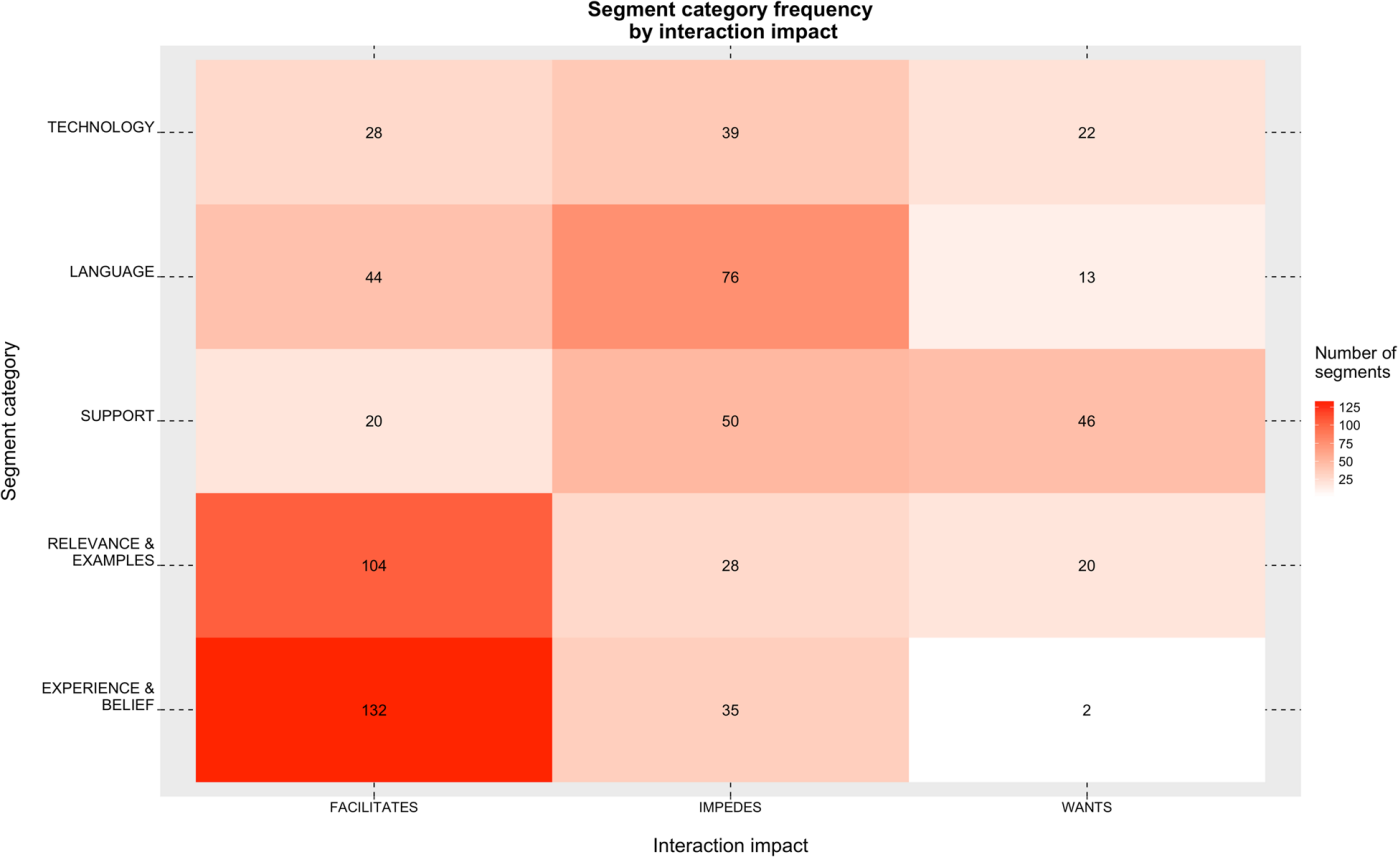
Frequency of segment categories, grouped by type of interaction impact. On this heatmap, each coded segment is counted equally and is only listed in one group. The interaction impact represents whether the segment was coded positively (facilitates), negatively (impedes), or as a recommendation for improvement (wants). The number listed within each group is the total number of coded segments in that group

A total of 550 min of transcript were segmented (mean = 92 min/session, range 60–120 min). Each transcript contained an average of 172 segments, and there were a total of 1032 coded segments. Figure 2 summarizes the frequency of usability factor interactions. Of the total coded segments, 532 (52 %) were associated with facilitating interactions, and 347 (34 %) were associated with impeding interactions. One hundred fifty three segments (15 %) were coded as a recommendation for a new feature or identification of a lacking feature (“wants”). The majority of segments (826, 80 %) were related to tool/user interactions, with most (447, 54 %) representing facilitating interactions. Five main themes of recurring factor interactions which influenced participants’ use of MOVE!23 were identified during analysis of the transcripts (see Fig. 3) and are described below.

### Technical ability and technology preferences

Comments associated with interaction between the user and the computer, both elements of the context of use, suggested that lack of experience with technology was a significant impediment to effective use of MOVE!23 for three (P1,P4,S1) of the six participants. Two (P1,P4) had very limited experience using computer hardware including laptops. They had difficulty physically interacting with a trackpad or mouse and relied heavily on the facilitator for support in task completion. In one instance, the participant asked the facilitator for a demonstration. A third participant (S1), though experienced, found that use of the scrolling feature of the unfamiliar touchpad was confusing.

Three participants (P1,P2,S1) indicated routine use of or preference for a specific hardware platform including tablets and smartphones. One participant (P1) stated: “[Using the mouse] is okay. At home I use a tablet and my husband is the one that has been using the laptop.” Another (P2) reported using weight management software already available on smartphones: “I use one application when I have my smart phone…It shows restaurant food, like the calories, different foods, and it shows if you do like an exercise, you can say how long you did it for and it records how many calories you burned.”

Statements made by four participants (P1,P2,S1,S2) suggested that they desired having the tool readily accessible in different settings. One participant (S1) stated: “I think [weight management software] is a good idea because I’m always on my phone and having the app, I would want to open it and continue using it. On the computer, sometimes I don’t feel like turning it on.” The advantages of printed handouts were also noted. One participant (P2) reported: “I would say [I prefer the patient report on] paper. So it could be with the person and help them do their weight loss.” Some preferred mixed media, with one participant (S1) concluding: “Maybe having both options because I know some people are more comfortable having paper in front of them rather than looking at it. But at the same time, you can also lose paper, and having access to it online would be helpful.”

### Language confusion and ambiguity

All six participants (P1,P2,P3,P4,S1,S2) encountered instances when MOVE!23 ‘s usability was impeded by unfamiliar or ambiguous language . When responding to a survey item, one participant (S2) had difficulty understanding “out of control” when referring to binge eating. Two participants (P1,P3), confused by unfamiliar health terminology, had difficulty understanding the term “active infection.” One participant (P1) confused the term “eating disorder,” which is commonly meant in medical contexts to invoke diseases such as anorexia or bulimia, with having poor eating habits noting, “I have a bad eating disorder because I would eat at night and now before I go to bed you see me eating a lot of sweets.” This participant (P1) also had difficulty with the terms “flaws” and “barriers” and needed extra time to define them in the context of MOVE!23.

At times, comparative language indicating size or degree led to ambiguity and confusion, hampering effective and efficient interaction with MOVE!23. Four participants (P1,P3,P4,S2) indicated uncertainty when responding to questions using the terms “moderate” and “vigorous” to classify their exercise habits. When answering a survey item indicating satisfaction with one’s appearance, participants (P4,S2) had trouble identifying with and differentiating between different response levels including “Moderately dissatisfied”, “Neither satisfied nor dissatisfied” and “Very unsatisfied.” Likewise, when asked to choose from a list of barriers to weight loss, two participants (P3,S2) noted difficulty interpreting and quantifying “too much stress,” in the response choices. One participant noted, “Some people probably, it’s normal for them to have this kind of schedule, so it’s not too much stress for them.”

One participant (P1), when asked during the retrospective interview to identify the most important change that could be made to MOVE!23, stated: “If only they could write it for people to understand it more.” One participant (P4) also suggested that language could impede satisfaction, noting that complex language might “overwhelm” and “discourage” users.

Although these participants were bilingual, none cited English language proficiency as cause for their confusion with the tool. No participants spontaneously suggested translating the tool into Spanish, although when specifically asked during the post-session interview, two (S1,S2) participants indicated that translation might be helpful to others.

### Supportive tool design and facilitator guidance

Participants responded favorably when features of MOVE!23 guided and supported tool use. Simple design features including highlighting and underlining helped to focus attention and facilitate interaction. One participant noted, “This is highlighted which is good because [the assessment tool] might start getting a little tedious and this helps me focus,” but suggested that it was not utilized often enough: “I know it’s very minor, but maybe bolding key words…to make it stand out [might help].” Five participants (P1,P2,P3,S1,S2) suggested that the addition of pictures could enhance tool use, motivation, and satisfaction but generally only when directly asked by the facilitator (“Just a little icon or something to brighten things up because after a while it’s just words and words.”)

At times the tool failed to effectively provide sufficiently comprehensive instructions and feedback for some participants (P1,P2,P3,P4,S2). Utterances such as, “And I click here? That’s all I do?” indicated participants’ uncertainty about their actions, seeking affirmation before committing to them.

Five participants (P1,P3,P4,S1,S2) had varying degrees of difficulty expressing the frequency of their activities and behaviors in specific units as required by a survey item. For example, two participants (P3,S1) had difficulty indicating, on a numeric scale (0 to 21), the total number of times per week including breakfast lunch and dinner, they ate a meal prepared at a restaurant. Participant P1 noted:

RESPONDENT: “Breakfast, lunch and 7 days a week, okay. A total of 21. Okay, so that’s fine. So, how many times a week do you eat [at the] restaurant? So I have to add. It’s like adding to 21 then, right? Am I understanding this? The right way? I don’t think so.”

One participant (S2) who completed the item unaided indicated that she had to “read it twice” and that it might be “confusing.” Two participants (P1,P3) relied on the facilitator for assistance with the task. When the task was redefined in familiar units or broken down into steps, participant satisfaction improved. When the facilitator redefined the units used in the question to more closely reflect a participants’ experience, she (P1) noted:

RESPONDENT: I mean you explain it to me and it makes sense now. I don’t know why I had difficulty understanding it.

### Relevant examples

Statements made by all six (P1,P2,P3,P4,S1,S2) of the participants suggested that inclusion of specific examples and definitions in both the questionnaire and patient report made it easier for them to interact with MOVE!23 and increased their satisfaction with the task. When completing the questionnaire four participants’ (P1,P2,P3,P4) utterances referred explicitly to examples while formulating responses. This was further evident in comments like (S2) “I think the definitions are nice… I didn’t know vacuuming was a moderate physical activity…without the examples, it would be confusing.”

Five participants (P1,P2,P4,S1,S2) made statements during review of the patient reports suggesting that examples and “tips” allowed them to relate to the recommendations and made them actionable. One (S2) noted, “Emotions and your weight. Okay. Yeah I like this handout, there’s a lot of useful tips that can be done by anybody.” Another liked the individual relevance of the tailored advice, indicating: “I feel that…I don’t ask enough. Like how [food] is prepared. If I have it [the handout] with me at a restaurant I would try to see how it’s prepared.”

Three participants (P2,S1,S2) suggested that the inclusion of more examples and “tips” would enhance the tool. One participant stated, “The other thing, having an example of how you can use your family or friends for support…if the whole family also follows the same diet, then it would help them achieve.” Another participant wanted more specific examples of the consequences of eating unhealthy foods: “I think [the handout] should have junk food to show them…this is what it’s going to do to your health in the future years, and the good food and how it will help improve your health.”

### Personal experience

All six participants (P1,P2,P3,P4,S1,S2) referred to personal experiences as they interacted with MOVE!23’s survey and patient reports. Moreover, recollections of personal experiences were used to reason through questions and formulate responses.

References to personal experiences varied in their level of detail. Participants (P1,P4,S2) responded to some survey items with little hesitation, making only brief references to personal experiences. At times content resonated more deeply with participants leading to more elaborate comments. When asked, “How satisfied are you with the appearance of your body?” one participant (P1) stated, “I don’t look good while my belly is sticking out, and I feel yucky. I’m embarrassed of my body…My husband told me I was fat…We’ve been married for more than 40 years.”

Participants (P1,P3,P4) actively sought personal experiences to help them comprehend unfamiliar or ambiguous language. Confused by the meaning of “Active infections of any kind” in a question about chronic medical problems, one participant (P1) tried to recall an experience that would support her response: “I get urine infections, so that means I’m supposed to write it here?…Not right now, but that’s what I get a lot.” Although she found an example of “infection,” the fit was imperfect and led to uncertainty and a request for support. In response to the question “Have you tried to lose weight in the past?,” another participant (P3) reflected on past attempts at weight loss to overcome confusion about the term “tried” stating, “No I haven’t tried. I just stop eating certain things that I know that’s the reason why I’m gaining weight. So eventually I hope that I will lose weight but it’s not like I’m thinking about it.”

The selection and acceptance of goal setting guidance was also influenced by personal experience and beliefs (P1,P2,P4,S2 ). One participant (P4) was attracted to advice related to medical conditions and activity noting, “Sometimes I get real bad pains and arthritis, sometimes when it grabs me here or in my knees…the arthritis is so bad.” Another (P1) initially questioned the recommendation to substitute diet soda for other calorie filled beverages because in the past she had found diet soda to taste sweeter than non-diet soda.

## Discussion

We undertook this study to determine if the MOVE!23 tool could be repurposed for use in the Latina population. We aimed to determine how specific interactions between the tool and other contextual elements facilitated or impeded its use, and determine what kinds of changes to the tool and/or its context might support usability. Review of the qualitative data provided insight into several factors which influenced participants’ ability to complete the questionnaire and review the tailored patient report with efficiency, effectiveness and satisfaction. Participants’ behavior and utterances around interactions, especially when they turned to the facilitator for support, provide insight into both the obstacles faced by the participants as well as the types of interventions that might help to resolve them. While many usability impediments derived directly from the interaction between the user and the tool, many also were a byproduct of the specific context of use. Given our findings we suggest several design considerations that may inform the adaptation of MOVE!23 and the design of eHealth applications in general (see Table 3).

**Table 3:** Potential MOVE!23 tool adaptations

Interaction	Feedback/Rationale	Proposed changes
Tool-User	Aid in interpreting questions and advice output^a^	- Replace complex and ambiguous survey language - Consistently provide definitions for complex or ambiguous terms
Tool-User	Stimulate recall of relevant behaviors^a^ Promote mapping behaviors to response	- Incorporate relevant examples into the questionnaire
Tool-User	Prevent estimation errors and promote mapping behaviors to response	- Allow users to respond to questions in units that match their behaviors (i.e. per week, per month)
Tool-User	Increase personal relevance of communication^a^	- Allow the user to further personalize goals by selecting and prioritizing advice. - Include actionable examples in goal-setting advice
User-Context	Deliver contextualized and actionable communication	- Allow the user to access goal-setting advice at times and places, and in a form that supports action
Tool-User	Provide adaptive task support^a^	- Ensure that support of a peers (*promotoras*) or healthcare professionals are available when completing the questionnaire and reviewing the patient report
User-Context	Provide adaptive technology support^a^	- Provide direct and immediate support for the use of technology when using MOVE!23 in clinical settings

^a^can also be addressed through support from *promotoras* or other peer/lay health coaches

Language confusion and ambiguity had a broad impact on interaction with MOVE!23. All six participants encountered unfamiliar or unclear language which impeded use of the tool. Participants would often focus on specific familiar words or concepts used in the materials to find personal experiences that best fit the question or advice to complete the task. Ultimately they either tenuously settled for a choice that seemed appropriate, or asked for support. Familiar language and the use of explanations, examples, definitions and visual cues supported participants understanding of the content, enhancing ease of use and satisfaction.

As Schwartz & Oyserman have indicated [36], when responding to a behavior question, respondents must understand the meaning of the question, identify relevant personal experiences that relate to the question, perform estimation or inference operations to make their recollections fit the response format, map their recollections to the available response alternatives, and “edit” their responses to project the desired social image. The characteristics of survey questions can influence this cognitive process and the accuracy of behavioral self-reporting. Our observations are consistent with this perspective, and exemplify how language complexity and ambiguity, and perceived content relevance influence a respondent’s ability to engage in behavioral self-evaluation.

To ensure comprehension, eHealth tools should use common and familiar language where possible and, where unfamiliar terminology is necessary, provide definitions in context. When asking users to express behaviors quantitatively, designers should be aware that the characteristics of their question may not readily correspond to the experiences of their users. Our study supports that whenever possible, common, real-world examples should be incorporated into the application to facilitate comprehension, acceptance, and task accomplishment.

Our findings also suggest that when eliciting or presenting quantitative data, designers should recognize that the user may need to translate terms in order to reconcile the units of measure used by application content with those of their personal experiences. Five participants in this study had varying degrees of difficulty interacting effectively with content involving unit translation. We suggest that applications segment tasks involving such translation into more manageable steps, explicitly guide the user through the steps, and automate all or part the translation process where possible.

Our findings further suggest that users value the availability of application features and content that can be readily used in support of their daily activities. The designer, however, should carefully consider the technologies available to and the technical abilities of their target population and select delivery media accordingly, lest they exclude those who might otherwise benefit from the intervention.

The nature and variability of the usability difficulties experienced by participants pose a particular challenge to the MOVE!23. Our findings show that our participants value and seek support, relevancy, and a means to connect the assessment tool to their personal experiences. However, given the diversity of cultures and experiences of Latina patients within the US [27], relying on technology adaptation alone may not address all possible barriers to use and acceptance by Latina patients. We suggest that to address this challenge, there may be merit in augmenting the context of use, rather than the tool, to promote overall task accomplishment. For instance, other studies of targeted interventions for Latina patients have used *promotoras*, lay community health workers from local communities, to assist patients in health and lifestyle interventions [36–38]. The use of trained *promotoras* with knowledge of MOVE!23 may provide a flexible and responsive way to support Latina patients in their use of the tool. Additionally, they can assist in the formulation and execution of a weight management strategy in conjunction with the tool. This has the potential to increase efficiency, effectiveness, and satisfaction. In evaluating the usability of an application, we recommend that designers not lose sight of the larger user experience and consider changes to elements of the context of use, where appropriate, to enhance users’ capabilities and application usability.

### Limitations

The most notable limitation of this study is its small sample size. There is no universally accepted standard sample size number for usability studies [39], and the appropriate sample size for such studies has been debated [40–43]. Some researchers have suggested that small sample sizes are appropriate because they allow for in-depth analyses to occur [21, 44] while others have indicated that small sample problem discovery rates may be overstated, recommending other approaches to sample size estimation [39, 42, 45–47]. Although the sample size of this study may not be sufficient to identify all usability issues, the study was formative in its approach [48], seeking to detect and categorize the most common and severe usability problems occurring in the target population rather than provide an exhaustive quantitative assessment of its usability. To this end, we believe that the sample size, and the depth and richness of our data were sufficient to uncover representative and frequently occurring usability challenges facing participants.

Participants were also drawn from a pool of English-speaking Latinas living in a large metropolitan area that were predominantly of Puerto Rican descent. The study, therefore, may not reflect the preferences and challenges found among Latinas who are recent immigrants, from other cultural backgrounds, or living in suburban or rural communities.

We also acknowledge that there are other structured methods of usability inspection [49, 50], such as the classic heuristic method suggested by Nielsen & Molich [51], which may have uncovered additional application design issues or permitted a more formal and quantifiable rating of usability. We note, however that while our study did not use the well-known usability heuristics discussed by Nielsen [52], our observations and findings are still consistent with it. For example, the “Match between system and real world” heuristic suggests that language and concepts used by a tool be familiar to the target user. Our observations of the impact of language relevance and clarity among the Latina users exemplifies this principle. In addition, by engaging representative participants from the target population rather than design experts, we gained contextual insight into the mechanism underlying the user–tool impediment (ex. ambiguous comparative language, health literacy, etc.). Nielsen recognizes the limitations of relying solely on heuristic evaluators, and acknowledges the importance of user testing to fully understand tool use in a specific context [53].

Finally, although efforts were made to insure that the facilitators’ interventions during the protocol were neutral, reminders and instructions used during the usability sessions may have disrupted participants’ thought processes or influenced participants’ responses. The necessity for neutral think-aloud practices in usability studies are described by Ericsson and Simon [32], and the consequences of departing from neutral prompts has been noted elsewhere [54, 55].

### Applications & future directions

We proposed several adaptations to the MOVE! assessment tool based on the findings from this study (see Table 3), and of note, a brief summary of our initial findings was submitted to the former National Program Director for Weight Management at the VA National Center for Health Promotion and Disease Prevention (NCP), who has been key in expanding the MOVE! program. MOVE!23 was updated and the revised version (MOVE!11) released in Spring 2014 incorporated a few of our recommendations. While many general recommendations have been incorporated in the revised MOVE!11 tool, it remains a product targeted largely at the needs and characteristics of the broader Veteran patient population. Additional iterative studies of the evolving MOVE! assessment tool and its implementation in the Latina community with larger groups of participants are required to ensure that enhancements address the challenges that have been identified, and to uncover additional, less frequently occurring problems. Future research should also include a more diverse sample of Latinas, whose beliefs, experiences and cultural characteristics may not have been represented in our study. Finally, a more formal assessment of language proficiency (reading, writing, speaking, listening) should be incorporated into future studies to help better understand the impact of bilingual English language proficiency, language ambiguity, health literacy, and general education levels on tool usability.

## Conclusions

We conducted this qualitative usability study in order to assess Latina users’ reaction to and interactions with the existing MOVE!23 assessment tool (originally developed for use among Veterans) and determine if it could be adapted for use with Latina patients. We found that the ability of Latina participants to successfully use the tool was influenced by the interaction of individual characteristics with those of the tool and other contextual factors. We also identified both tool-specific and context-related changes to overcome barriers and enhance use of MOVE!23 in this population. Those developing eHealth or technology-assisted interventions may benefit from using a similar methodological approach if adapting existing eHealth tools for use in different populations.
